# Novel Conservative Approach to High Surgical Risk Frail Proximal Femur Fractures

**DOI:** 10.1155/2020/8847080

**Published:** 2020-06-22

**Authors:** Alexander Koizia, Ala Abuown, Julie Vowles, Damien Smith, Louis J. Koizia

**Affiliations:** ^1^East Suffolk and North Essex NHS Foundation Trust, UK; ^2^Imperial College Healthcare NHS Trust, UK; ^3^The Hillingdon Hospitals NHS Foundation Trust, UK; ^4^Cutrale Perioperative and Ageing Research Group, Department of Bioengineering, Imperial College London, UK

## Abstract

One of the major impacts following a neck of femur fracture is pain. Most patients (nearly all) undergo an operation. This usually includes the frailest terminal patients and deemed a palliative procedure to reduce ongoing pain. The operation comes with risks and can reduce life expectancy in these patients and result in prolonged hospital admission, delirium, and postoperative complications. This case highlights a novel approach to managing the frailest end-of-life patients that does not require them to undergo a conventional operation. The case resulted in a quick discharge from hospital and for the patient and family to maximise the time out of hospital, with a reduced analgesic burden and a peaceful passing away. We feel that this could be an alternative, more humane option for such patients.

## 1. Introduction

Today, the vast majority of patients are managed following a hip fracture with an operation; this stems from the greater mortality in those patients not operated, including the frailest cohort [1, 2] . In addition, the operation provides fixation of the underlying fracture and reduces pain overall. Therefore, a hip operation following a hip fracture reduces mortality, pain, and length of stay and increases mobility [3–5] . Some cases that are deemed too high risk for an operation can be managed conservatively, but this comes with issues of inadequate pain relief, patients being immobilised for a prolonged period, and prolonged hospital stay. Very little literature is available on alternative approaches to these patients that are unable or deemed unfit for an operation.

## 2. Case Report

### 2.1. History

An 89-year-old gentleman was admitted to a district general hospital with right groin and thigh pain. He was known to suffer from Alzheimer's dementia, osteoarthritis, previous proximal femoral fracture with dynamic hip screw (DHS), and severe aortic stenosis. He was living in the local area in a nursing home, requiring care with all activities of daily living, spending the vast majority of the day in bed. He was able to transfer with assistance of two into the chair but unable to walk prior to admission.

On admission, he was found to have an externally rotated right hip and pain on movement of the hip. His abbreviated mental test score (AMTS) on admission was zero, and that was felt to be his baseline. On auscultation of the precordium, a very quiet second heart sound was heard with an additional ejection systolic murmur present.

### 2.2. Investigations

Basic blood tests showed a haemoglobin of 115, white cell count of 11.9, C-reactive protein of 130, sodium of 137, potassium of 3.8, urea of 8.3, and creatinine of 113.

Pelvic X-ray was reported to identify generalised osteopenia, left DHS, and displaced surgical neck fracture of the right femur. An urgent echocardiogram identified an ejection fracture of 60% and a severely stenosed aortic valve with an area of 0.8 cm^2^ and a gradient of 71 mmHg.

Based on his cardiac, cognitive, and functional status, the decision was made that his outcome was extremely poor and he was deemed not to be a surgical candidate.

### 2.3. Procedure

As a result of him not undergoing a conventional operation and him being managed conservatively, we felt that consideration for a phenol nerve block would aid pain relief and assist in being able to discharge him into the community for end-of-life care. As the patient lacked capacity to consent for a proposed intrathecal phenol injection for intractable hip pain, secondary to the fracture, it was deemed in his best interest to proceed. The plan was discussed and agreed with the next of kin and multidisciplinary team.

The procedure was performed in the anaesthetic room in aseptic conditions (gown, sterile gloves, hat and mask). The patient was attached to full monitoring with supplemental oxygen given. Intravenous sedation was given in preparation for the block in the form of 1 milligram of midazolam with 5 milligrams of ketamine.

The patient was placed in a right lateral position (bad side down). 0.5% chlorhexidine was applied to the skin. The L4 level and spinous process were palpated using Tuffier's line. A 25G needle was inserted 2 cm right lateral to the spinous process infiltrating 4 millilitres of 1% lidocaine at a 45′ angle aiming for a lumbar interlaminar space via a paramedian approach.

A 20G Quincke spinal needle was passed until cerebrospinal fluid (CSF) was observed. 1 millitre of 0.5% heavy bupivacaine was injected; then, the patient was observed for 5 minutes for any cardiovascular instability. Following this, 1 millilitre of 5% phenol in glycerol was injected via the Quincke needle. The spinal needle was flushed with 0.5 millilitres of 1% lidocaine, and CSF was observed.

Dressing was applied, and the patient was placed in the recovery unit and remained in a right lateral position for 30 minutes.

### 2.4. Postprocedure

Following the procedure, the patient was not complaining of any pain in his hip (the Abbey Pain Scale improved) and we were able to rotate the hip without any tenderness. He was discharged back to his nursing home with the community palliative care team. He remained pain free and required limited medication for symptom control and passed away four weeks after the neck of femur fracture.

## 3. Discussion

We report the successful use of targeted phenol neurolysis in an end-of-life patient with a conservatively managed hip fracture. This intervention left him pain free, with a short admission to hospital without high-risk surgery or the prolonged use of opioids.

Early operative repair is the standard management for hip fractures in almost all cases. NICE guidance recommends “if a hip fracture complicated or precipitates terminal illness, the MDT should still consider the role of surgery as part of a palliative care approach” [6]. Of the 76,000 hip fractures in the UK each year, only 2.2% are treated nonoperatively [7]. These are largely patients who have been deemed to have unacceptably high risk of surgical complications or perioperative death. Options for managing this small subset of patients are extremely limited. In 20 years, the population of over 85s is predicted to double from 1.6 million to 3.2 million [8]. More very elderly patients will present with hip fractures who are not surgical candidates. There is an ever increasing and unmet clinical need for novel approaches to manage this patient group.

These patients have poor outcomes. One meta-analysis reported an unadjusted odds ratio of 3.95 for 30-day mortality in patients managed nonoperatively compared to the operated group [9]. Gregory et al. (2010) found that excess deaths in this 30-day period were due to the underlying medical conditions that had made them unsuitable for surgery [5]. They concluded that these patients' outcomes would not have been improved by surgery.

Without surgical fixation, achieving adequate pain control presents a significant challenge. Nonoperative management is currently dependent on opiate analgesics, which are likely needed for a prolonged period of time. This is in an already frail population at higher risk of side effects such as sedation, acute delirium, worsening cognitive impairment, respiratory depression, nausea, and constipation.

Peripheral nerve catheters are widely used to good effect in the acute management of hip fractures. In nonoperatively managed NOFs, Rashidifard et al. (2019) demonstrated that continuous peripheral pain catheters decreased visual analogue scale (VAS) scores of an average of 4.45 points after 24 hours [10]. 90% were able to increase their level of ambulation. However, these catheters have a life span of 2-3 days. Phenol neurolysis, as demonstrated in the case above, has the potential to produce similar benefits but with long-term effect.

Neurolysis involves the chemical or physical denervation of a nerve. Phenol induces Wallerian degeneration by causing separation of the myelin sheath from the axon and axonal oedema. Effects are produced in minutes and can last months to years depending on the target nerve. Phenol neurolysis was first described in a 1955 report by Maher, who administered phenol intrathecally for the relief of terminal cancer pain [11]. It was later adopted in the management of spasticity in the 1960s.

The use of phenol as a neurolytic agent has declined in recent decades due to concerns related to its possible serious adverse effects, including dysesthesias, flaccid paralysis, tissue necrosis, and systemic complications. However, modern techniques allow highly accurate delivery of phenol to the target nerve and favourable safety profiles [12].

Neurolysis for spasticity is aimed at preventing contractures, reducing pain, and improving overall functional status [13]. It is used in central nervous system disorders, including stroke, brain injury, and cerebral palsy. There are small-scale promising studies reviewing their efficacy and safety for this indication, Karri et al. (2020) reviewed a group of 185 patients who underwent phenol neurolysis for focal spasticity [14]. 84% of those treated reported a benefit, and promisingly, there were no major adverse events reported. Minor adverse events included pain (4%) and swelling/inflammation at the injection site (2.7%).

Phenol neurolysis has a good evidence base in some areas of palliative pain management; particularly patients with intractable pain refractory to high-dose opioids. Celiac plexus neurolysis (CPN) is well established in the treatment of visceral pain resulting from upper abdominal malignancy, mainly cases of pancreatic cancer. A Cochrane systematic review comparing CPN to conventional therapy found improvements in VAS, a significant decrease in opioid consumption, and fewer reported adverse side effects at four and eight weeks [15]. They reported no major complications related to the procedure. They concluded that CPN was a safe and effective option in the management of pancreatic cancer-related pain.

There is some evidence for the use of phenol neurolysis in the management of nonmalignant pain. Weksler et al. (2007) studied the efficacy and safety of phenol neurolysis in a heterogenous group of patients with intractable nonmalignant pain [16]. Participants had failed to achieve adequate pain control through other modalities. Various peripheral nerves were targeted, including intercostal and genitofemoral. 83% achieved good pain relief (VAS of 3 or less), with a mean VAS prior to treatment of 8.7 compared to 1.93 post treatment. No major adverse effects were reported. Minor complications included local haematoma (7%) and pain at the injection site (14%).

## 4. Conclusion

Phenol is an effective and relatively safe neurolytic agent which already has a good evidence base for use in malignant pain and spasticity. There are a small number of promising studies which support expanding its use for non-malignant pain. This case report presents a new clinical application of a treatment modality that has been used for decades. This intervention could have a particular value in terminal NOF fracture patients with poor prognosis and deemed not fit for surgical fixation. It provides a novel means of pain palliation in neck of femur fractures. In this case, phenol neurolysis was effective at reducing pain, reduced opioid use, and gave an alternative to high-risk surgery in a very frail patient.
